# Restoration of Life

**Published:** 1885-10

**Authors:** 


### Restoration of Life.

Dr. Richardson has started the question whether life may not be restored after actual death, and relates some facts that point to the answer as being in the affirmative. By combining artificial circulation with artificial respiration, a dog was restored to life an hour and five minutes after having been killed by an over-dose of chloroform, when the heart had become perfectly still and cold, and was passing into rigidity. Animals that have been killed by suffocation and partially dissected were brought to such a state of muscular irritability that the experiment was stopped for fear that they would return to conscious sentient life. Frogs poisoned by nitrite of amyl were restored after nine days of apparent death, in one case after signs of putrefactive change had commenced. The action of peroxide of hydrogen in reanimating the blood and restoring heat in a really dead body is quite startling. From these observations, Mr. W. Mattieu Williams thinks the conclusion is justified that "a drowned or suffocated man is not hopelessly dead so long as the bodily organs remain uninjured by violence or disease, and the blood remains sufficiently liquid to be set in motion artificially, and supplied with a little oxygen to start the chemical movements of life."

